# Radiofrequency-assisted transection of the pancreas vs stapler in distal pancreatectomy: a propensity score matched cohort analysis

**DOI:** 10.1038/s41598-022-11583-0

**Published:** 2022-05-06

**Authors:** E. Pueyo-Périz, C. Téllez-Marquès, A. Radosevic, O. Morató, L. Visa, L. Ilzarbe, E. Berjano, E. de Vicente, I. Poves, B. Ielpo, L. Grande, F. Burdío, P. Sánchez-Velázquez

**Affiliations:** 1 Division of Hepato-Biliary and Pancreatic Surgery, Department of Surgery, University Hospital del Mar-IMIM (Hospital del Mar Medical Research Institute), Passeig Maritim 25-29, Barcelona, Spain; 2 Department of Radiology, Hospital del Mar, Passeig Maritim 25-29, Barcelona, Spain; 3 Department of Oncology, Hospital del Mar-IMIM-CIBERONC, Passeig Maritim 25-29, Barcelona, Spain; 4 Department of Gastroenterology, Hospital del Mar, Passeig Maritim 25-29, Barcelona, Spain; 5 BioMIT, Department of Electronic Engineering, Universitat Politècnica de València, Valencia, Spain; 6 Department of Surgery, Hospital Sanchinarro, Madrid, Spain

**Keywords:** Gastrointestinal cancer, Gastroenterology

## Abstract

To demonstrate the efficacy of radiofrequency for pancreatic stump closure in reducing the incidence of postoperative pancreatic fistula (POPF) in distal pancreatectomy (DP) compared with mechanical transection methods. Despite all the different techniques of pancreatic stump closure proposed for DP, best practice for avoiding POPF remains an unresolved issue, with an incidence of up to 30% regardless of center volume or surgical expertise. DP was performed in a cohort of patients by applying radiofrequency to stump closure (RF Group) and compared with mechanical closure (Control Group). A propensity score (PS) matched cohort study was carried out to minimize bias from nonrandomized treatment assignment. Cohorts were matched by PS accounting for factors significantly associated with either undergoing RF transection or mechanical closure through logistic regression analysis. The primary end-point was the incidence of clinically relevant POPF (CR-POPF). Of 89 patients included in the whole cohort, 13 case patients from the RF-Group were 1:1 matched to 13 control patients. In both the first independent analysis of unmatched data and subsequent adjustment to the overall propensity score-matched cohort, a higher rate of CR-POPF in the Control Group compared with the RF-Group was detected (25.4% vs 5.3%, p = 0.049 and 53.8% vs 0%; p = 0.016 respectively). The RF Group showed better outcomes in terms of readmission rate (46.2% vs 0%, p = 0.031). No significant differences were observed in terms of mortality, major complications (30.8% vs 0%, p = 0.063) or length of hospital stay (5.7 vs 5.2 days, p = 0.89). Findings suggest that the RF-assisted technique is more efficacious in reducing CR-POPF than mechanical pancreatic stump closure.

## Introduction

Distal pancreatectomy (DP) is the gold standard surgical procedure for most benign or malignant lesions in the body or tail of the pancreas, which is defined as any resection of the pancreas beyond the left aspect of the superior mesenteric vein/portal vein trunk^1–3^. Although this surgery is performed less often than pancreaticoduodenectomy, improvements in diagnostic imaging techniques have resulted in an expansion of DP indications. In recent years, the mortality rate following DP has decreased dramatically to between 0 and 5%^2,3^ in high-volume centers due to advances in surgical techniques and especially to improvements in perioperative care^3^. Despite this reduction in the mortality rate, morbidity remains high mostly as a result of complications related to the pancreatic transection and the development of postoperative pancreatic fistula (POPF), which is associated with intra-abdominal abscesses, sepsis, respiratory complications and hemorrhage among others^1^, and which prolong hospital stays and raise costs for specialized treatment, including revision surgery and drainage^4^. The fundamental shortcoming in outcome assessment after DP is the lack of genuine outcome data, therefore, since relevant studies report CR-POPF ranging between 10 and 40%^1,2^, a fistula rate of around 30% is generally accepted.

Despite efforts to reduce the incidence of POPF through the use of many different stump closure methods such as staplers, hand-sewn closure^5^, biological sealants^6,7^ or even patches of fatty tissue applied to the pancreatic stump, the incidence of POPF remains a clinically relevant issue and has been relatively stable over recent decades, even with the introduction of minimally invasive surgery^2,8^, with no technique demonstrating better results than others^3,5–7^. In fact, there is considerable variability in clinical practice worldwide reflecting the lack of solid evidence on the benefit of any given strategy, indicating that there are still underlying areas for improvement and that new techniques are needed in order to improve results after DP^9^. One of these emerging techniques is based on using radiofrequency (RF) energy^10,11^. During the application of RF, tissue heats up gradually, reaching above 60 °C and causes denaturation of cell membrane proteins, cytoplasm, mitochondrial enzymes, and nucleic acids, known as coagulative necrosis^12,13^, and thus this latter one could prevent POPF occurrence by eliciting fibrosis and collagen shrinkage^14–16^. Some experimental studies have demonstrated that RF-based DP improves the sealing efficiency of the main and secondary pancreatic ducts and reduces the incidence of POPF. Although literature on the issue is limited^17–19^, recent studies indicate notable reductions in the POPF rate when a RF device is used at the parenchyma transection plane^15–17^. One important clinical attempt was an unfinished RCT performed at the Mayo Clinic in Rochester, MN (NCT01051856) in which a RF device (TissueLink Medical, Dover, NH, USA) was compared with stapling. However, the trial was terminated early due to poor recruitment in 2014.

In absence of RCTs in this area, propensity score matching analyses provide evidence that minimizes bias from non-randomized treatment assignment and allows comparison of otherwise non-comparable cohorts. Applying this methodology to our study, we evaluate an innovative concept for pancreatic parenchymal transection, which has proved reliable and which background research^20,21^ indicates to be a better method of closing the pancreas remnant to reduce POPF than other standard techniques.

## Materials and methods

We performed a retrospective propensity scored matched cohort study comparing DP with RF closure of pancreas stump (RF Group) to standard DP conducted with other transection methods (Control Group). Ethical approval was obtained from the Institutional Review Board at Hospital Universitario del Mar (No. 2020/9390/I). All patients singed an informed consent to participate in the study. The study was performed in accordance with relevant named guidelines and regulations.

### Patient eligibility and data collection

All consecutive patients undergoing elective distal pancreatectomy in our institution between 2006 and 2019 were retrospectively included in the study. All data proceed from a prospective electronic database audited and checked for completeness by 2 investigators (EPP and CTM). Inclusion criteria were DP conducted in adults for any solid or cystic lesion. Stapler or other mechanical transection methods such as LigaSure were employed in the control Group, while in the RF Group, a radiofrequency device was exclusively used. Exclusion criteria were patients with insufficient baseline data or missing primary outcome data.

Included cases in the RF Group were all patients undergoing elective DP and performed by three surgeons who had already reached their learning curve. The technique was conducted in a standardized fashion with either open or laparoscopic approaches. After examination of the abdominal cavity, the gastrocolic ligament was divided to allow correct visualization of the upper border of the pancreatic gland and the course of the splenic vessels. Routine dissection of splenic vessels and encircling the splenic artery was prophylactically conducted in case a hemorrhage occurred. The position of the pancreas division line was selected in the proximal normal pancreas depending on the placement of the lesion and intraoperatively guided by ultrasonography to ensure correct margins. In all cases, pancreatic transection was performed in the RF group with a 10-mm diameter version of the Coolingbis device (Apeiron Medical, Valencia, Spain). By applying the device and moving it back over the surface of the parenchyma, the blunt part of the device coagulates the tissue and the blade part cuts through the portion of tissue coagulated^17,20^. Splenic preservation was conducted in patients with benign lesions or borderline tumors (e.g., cystic neoplasm), but not for pancreatic adenocarcinoma. In those specific cases, a Radical Antegrade Modular Pancreatosplenectomy (RAMPS) was performed.

**Figure 1:**
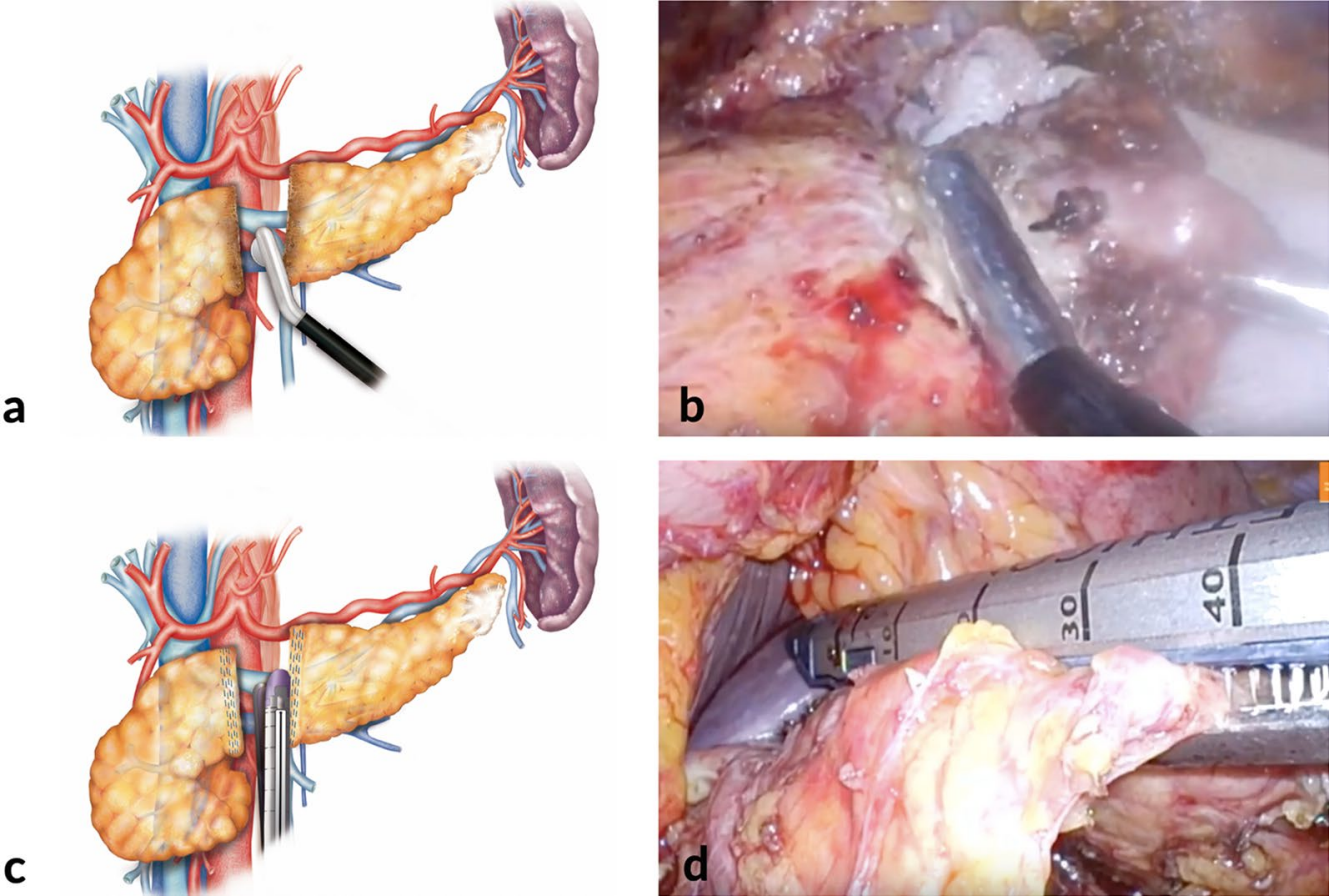
Pancreatic transection with the two different scenarios that have been described in this study. The upper figures (**a**,**b**) show representative (left) and original (right) methods of pancreatic transection mediated by RF. In contrast, the lower figures display the closure with a linear stapling device.

Included patients in the Control Group were all consecutive patients undergoing standard DP. The surgical procedure was performed in essentially the same way as stated previously in the RF Group except for the pancreatic transection, which was carried out mostly with a stapler. Differences between these techniques are outlined in Fig. 1.

### Primary and secondary end-points

The primary end-point was comparison of the clinically relevant postoperative pancreatic fistula (CR-POPF) between the two study groups, graded using the updated International Study Group on Pancreatic Fistula classification^8^. Secondary endpoints included 90-day postoperative mortality and complications recorded and monitored until 90 days after the operation. All surgical complications were graded using the Clavien–Dindo classification^22^, which categorizes the complication according to received treatment, and the CCI, a value which measures overall cumulative morbidity on a scale from 0 (no complications) to 100 (death), which was applied to cover the total number of complications by severity for each patient^23^. Complications were reported as major complications, i.e., Clavien–Dindo ≥ IIIa or minor complications, corresponding to Clavien–Dindo ≤ 2.

Within postoperative outcomes, special attention was paid to radiological evaluation of the transection zone after surgery. Computer tomography at 1-month and 1-year postoperative was assessed to evaluate the remnant necrosis at the transection zone, measured as the larger of the two orthogonal diameters, to compare between-group differences.

### Statistical analyses. Propensity score matching

With the aim of minimizing the differences and avoiding potential confounders between groups, propensity score (PS) matching was applied according to Lonjon et al.^24^ recommendations. The PS is defined for each participant as the probability of receiving the treatment, given baseline covariates. With the assumption of no unmeasured confounders, a treated and an untreated patient with the same PS can be considered as though they had been randomly assigned to each group.

Accordingly, an individual’s PS was calculated through logistic regression modeling based on the following covariates: age, American Society of Anesthesiologists (ASA) classification, body mass index (BMI) and type of pancreas resection (either distal pancreatectomy or RAMPS). Those covariates were selected as well-known factors that might influence the occurrence of CR-POPF and, most importantly, the surgeon’s tendency to choose one technique or another mainly based on the thickness of the pancreas or its proximity to the intrapancreatic common bile duct.

The surgeon tends to avoid the use of staplers in thick pancreas, which are more likely to crush when the stapler is closing or in the tail of the pancreas where it is bulkier and offers difficulties to apply a stapler. As such, these baseline characteristics could affect surgical outcomes. As there are different modalities in calculating a PS, we chose to do PS matching, i.e., matching participants with identical or similar PS with a 1:1 ratio without replacement and a standard caliper width of 0.3. The area under the curve (AUC) of the model was 0.847, which indicated a good goodness of fit.

To assess the validity of our PS matching, data analysis was subsequently divided into two phases. First, we analyzed the row data without matching PS and applying pairwise comparisons of the subsequent endpoint variables with Fisher’s exact test for categorical variables, and the Mann–Whitney *U* test for continuous variables. Following 1:1 matching of the cases (RF-Group) with the controls (Control-Group), comparability was assessed between propensity score-matched cohorts using McNemar’s test for paired categorical variables, while continuous outcomes used the paired *t* test or Wilcoxon rank-sum test. Categorical data were reported as proportions and continuous data as mean and standard deviation (SD). Tests were considered statistically significant at a 2-sided p < 0.05. All confidence intervals (CI) were 95%. All data were handled and analyzed using IBM SPSS Statistics for Windows version 25.0 (IBM, Armonk, NY, USA).

## Results

### Baseline characteristics

**Figure 2:**
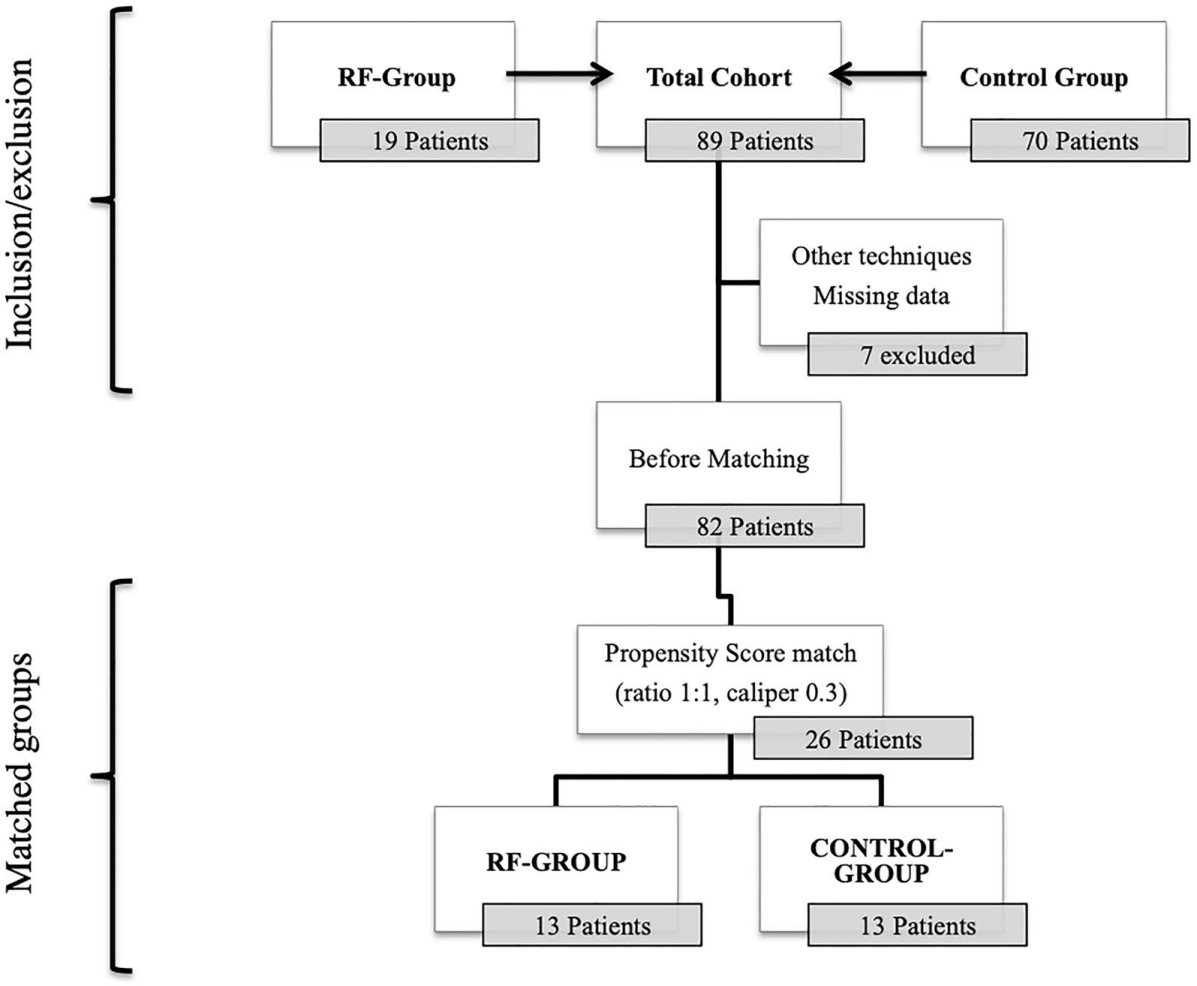
Flow chart of the study.

A total of 89 patients underwent DP in our institution between 2006 and 2019. Seven patients were excluded owing to inaccurate or missing data, or due to the use of other techniques. The remaining 82 patients underwent PS matching; 19 in the RF Group and 63 in the Control Group. Of all included patients, 13 patients in RF Group were 1:1 matched to 13 patients in Control Group using a caliper width of 0.3 of the logit of the propensity score (Fig. 2).

Almost all baseline variables were more balanced after PS matching (see Table 1). Differences between groups were notable regarding the location of the tumor; those in the Control Group had more tumors located on the neck/body with respect to the RF Group (61.9% vs 10.5%, p = 0.001) and the proportion of patients with malignancies in the histology was also higher in the Control Group before matching (see Table 1). Concerning the stump closure only 3 patients out of the initial 82 in control group had a closure different to stapler and within the matched cohort all patients from the control group were performed with stapler. Most of the patient’s demographic characteristics were comparable between groups.

**Table 1:** Comparison of baseline characteristics between RF-Group and Control Group before and after Propensity Score-Matching.

	With propensity score-matching			Without propensity score-matching		
	Control-Group	RF-Group	p	Control-Group	RF-Group	p
	(n: 63)	(n: 19)		(n: 13 )	(n: 13)	
Sex, female, n (%)	34 (54)	7 (36.8)	0.191	4 (30.8)	6 (46.2)	0.420
Age, mean (SD)	64.8 (14.9)	66.8 (14.9)	0.919	53.2 (18.9)	68.3 (14.1)	0.428
BMI, mean (SD)	25.5 (5.2)	27.4 (4.6)	0.755	25.2 (3.8)	27.4 (4.9)	
**ASA classification, n (%)**			0.303			0.239
I–II	35 (55.6)	8 (42.1)		8 (61.5)	5 (38.5)	
III–IV	28 (44.4)	11 (57.9)		5 (38.5)	8 (61.5)	
Malignancy, n (%)	45 (71.4)	10 (52.6)	0.126	5 (38.5)	7 (53.8)	0.431
**Localization, n (%)**			< 0.05*			1.0
Body-neck	39 (61.9)	2 (10.5)		4 (30.8)	4 (30.8)	
Tail	24 (38.1)	17 (89.5)		9 (69.2)	9 (69.2)	
**Histology, n (%)**			0.022*			0.082
Adenocarcinoma	32 (50.8)	6 (31.6)		2 (15.4)	5 (38.5)	
NET	9 (14.3)	4 (21.1)		2 (15.4)	2 (15.4)	
IPMN	10 (15.9)	9 (47.4)		3 (23.1)	6 (46.2)	
Chronic Pancreatitis	5 (7.9)	–		3 (23.1)	0	
Others	7 (11.1)	–		3 (23.1)	0	

*p-value for the difference in the independent analyses (row data).

Significant values are in bold.

### Assessment of primary and secondary outcomes

Concerning the primary end-point, the first independent analysis of unmatched data revealed a higher rate of CR-POPF in the Control Group compared with the RF Group (25.4% vs 5.3%, p = 0.049). In fact, after conducting the adjustment in the overall PS-matched cohort, this difference became even more striking as 53.8% of patients in Control Group developed CR-POPF vs none in the RF Group (53.8% vs 0%; p = 0.016) (Table 2).

**Table 2:** Postoperative outcomes for Clinically Relevant Pancreatic Fistula (CR-POPF) occurrence and secondary end-points comparing Propensity Score-Matched Cohorts RF-Group vs Control Group.

	Control-Group Pre-match	RF-Group Pre-match	p	Control-Group Post-match	RF-Group Post-match	p
	(n: 63)	(n: 19)		(n: 13)	(n:13)	
**Perioperative**						
Operative time, median (SD)	276 (71.4)	284 (70.9)	0.635	224 (60.8)	280 (109.5)	0.065^b^
**Type of resection, n (%)**			**< 0.05**			1.0
RAMPS	54 (85.7)	4 (21.1)		4 (30.8)	4 (30.8)	
Distal pancreatectomy	9 (14.3)	15 (78.9)		9 (69.2)	9 (69.2)	
Laparoscopic approach (%)	45 (71.4)	19 (100)	**0.031**	92.3	100	1.0
Hard/firm pancreas (%)	27.0	15.8	**0.021**	23.1	23.1	1.0
**Postoperative (90 days)**						
Morbidity, n (%)						
None or minor complications CD 0–II	51 (81.0)	18 (94.7)	0.173	9 (69.2)	13 (100)	0.063^a^
Major complications CD ≥ IIIA	12 (19.0)	1 (5.3)		4 (30.8)	0	
In-hospital mortality (%)	1.6	0	0.768	0	0	1.0
Pancreatic fistula grade B/C n, (%)	16 (25.4)	1 (5.3)	**0.049**	7 (53.8)	0	**0.016**^a^
Hemorrhage grade B/C, n (%)	1.6	0	0.768	0	0	1.0^a^
CCI (median)	0	0	1.0	20.9	0	0.026
Reoperation (%)	6.3	0	0.341	0	0	1.0^a^
Length of hospital stay, mean (SD)	8.9	4.7	**0.02**	5.7 (3.3)	5.2 (1.6)	0.89^b^
Readmission rate (%)	12 (19)	1 (5.3)	0.137	6 (46.2)	0	**0.031**^a^
Transfusion rate (%)	7 (11.1)	0	0.146	0	0	1.0^a^

^a^McNemar’s test, ^b^Wilcoxon test.

Significant values are in bold.

With regard to secondary end-points, no significant differences were observed in terms of in-hospital mortality, as none of the groups presented mortality in the matched cohort. RF Group showed fewer minor and major complications compared to the Control Group, although no statistical significance was achieved. The rates of grade B/C hemorrhage were comparable in both groups. Length of hospital stay was significantly increased in the Control Group in the individual analyses, but were similar after PS matching (5.7 vs 5.2 days, p = 0.89). The results also revealed better outcomes concerning readmission rate in the RF Group (46.2% vs 0%, p = 0.031). Laparoscopic approach was significantly higher in the control group before the PS adjustment, although no between-group differences were observed by matched cohort.

At 1-month postoperative follow-up, no significant differences were found in the maximum diameter of the residual necrotic zone at the transection zone (24.6 ± 23.0 mm in the RF-Group vs 31.3 ± 17.4 mm in the Control Group, p = 0.646). Nor were changes in maximum diameter observed at 1-year follow-up. (17.6 mm vs 24.2 mm, p = 0.398).

## Discussion

To our knowledge, this is the first study which compares, through exhaustive PS matching methodology, the efficacy of RF-assisted vs stapler pancreatectomy in terms of precluding the appearance of CR-POPF. Our analysis shows that this index complication after pancreatic surgery is significantly lower in cases of RF-assisted pancreas transection compared to the classical stapled pancreatectomy and reveals encouraging surgical outcomes. As mentioned previously, CR-POPF after DP remains an unresolved issue and despite the associated mortality, which is not very high if the complication is handled in a timely manner, it does represent an important source of further complications and a burden for the patient, caregivers and healthcare system. A recent multicenter, randomized clinical trial (DISPACT) failed to demonstrate greater efficacy associated with the mechanical stapler compared with the classical hand-sewn technique, with no differences observed between groups in the incidence of POPF. Fistula rates as high as 36% in mechanical stapler group, and 37% in the hand-sewn group were reported^5^, again highlighting the non-superiority of one transection technique over another to date.

Therefore, the pressing need to find an innovative surgical solution to the issue of POPF after DP is clear. RF energy has been employed for a long time in hepatic surgery and has demonstrated great effectiveness in achieving parenchymal hemostasis along with simultaneous significant reduction of intraoperative blood loss and biliary leak by inducing an obliterative effect on vascular and biliary structures on the liver^18,25^. Thus, it has been hypothesized in several experimental studies that it could have the same effect on the pancreas acini, producing a more severe macro and microscopic inflammatory response in the transection plane than occurs when a mechanical stapler is used^21^. Experimental studies showed promising outcomes in terms of both safety and feasibility^17,20^. The study by Dorcaratto et al.^17^ on a porcine laparoscopic model, which represent the backbone of this current work, revealed an interesting histological pattern in the transection line with a central area of coagulative necrosis surrounded by a large capsule of connective tissue up to 1.8 mm when applying RF-assisted transection whereas the group stapler showed soft thin fibrosis on the stapler line. The rationale behind this finding is that this connective tissue barrier might prevent the leakage of pancreatic juice, due to the firm, fibrotic condition of the pancreatic remnant stump, such as that found in patients with chronic pancreatitis, which are believed to be less likely to leak and all techniques in these patients seem to have a higher rate of success^3^. Even more importantly, these experimental data demonstrated a common pattern of shrinkage of the main and secondary pancreatic ducts with no signs of pancreatitis in the remnant pancreas.

In line with these experimental findings, two previous clinical studies showed a decrease in the rate of CR-POPF when applying RF energy. Fronza et al.^16^ reported their initial experience with RF energy for pancreatic transection using the Habib 4× device and even though it was a retrospective analysis involving only 14 patients, they showed a CR-POPF rate of 14.3% with only one surgical reintervention and no mortality, which suggests that RF energy is a safe and feasible method for pancreatic transection^16^. Shortly after, Blansfield et al.^15^ published a retrospective study with 62 patients in which 29 of them underwent RF-assisted pancreatic transection with the TissueLink device, showing a reduction in POPF from 36 to 10% in the experimental group^15^. Although both studies had important limitations, both concluded that RF-assisted transection might be a promising technique for reduction of CR-POPF after DP. Interestingly, another recent RCT comparing stapler transection with ultrasonic sealing, which might be similar to RF transection to some extent as both imply thermic mechanism of action, showed no differences in terms of CR-POPF. However, this study has major drawbacks. On one side, the authors intentionally excluded patients with bulky pancreas (i.e. preoperatively measured > 17 mm), which supposes an important selection bias for the technique assessment since these are the pancreas more likely to crush after the stapler closure and the ones, which might benefit most of a thermal transection. In our study we did not perform any preoperatively selection according to the pancreas stiffness, precisely to assess the efficacy of RF in any setting and try to avoid this selection bias. On the other hand, it has been demonstrated that ultrasonic dissectors, such as Harmonic enable a coagulation margin much thinner than the one performed by a RF device and it might not generate enough connective tissue barrier to preclude the collection of a pancreatic juice and therefore not be decisive to avoid CR-POPF.

In the first stages, however, the potential value of a novel procedure is still hampered by heterogeneity in the patient population and an absence of standardization of the technique, which precludes wide acceptance. To minimize bias and achieve a balanced exposure of groups at baseline, a propensity score-matching model was developed and we were able to demonstrate the greater efficacy of the RF-assisted technique in terms of decreasing CR-POPF not only in the individual row data analysis but also in the PS matching. Therefore, given the absence of any improvement in CR-POPF rates in the last decade, which remain around 30%, stapler closure may no longer be regarded as the current state-of-the-art technique for distal pancreatectomy^5,26^.

This study has however several limitations that warrant emphasis. First, the sample size is small, as there is a certain difficulty in recruiting patients for this surgical indication, and which becomes smaller still after PS matching to homogenize the study groups, which is one of the disadvantages of these type of studies. Moreover, the beginning of a new surgical technique usually faces many hurdles till it becomes widely accepted due to the initial doubts about its feasibility and safety, therefore the first preliminary reports published on this approach gathered no more than 20–30 patients. Although the study covers a long period of time, no significant variations in the technique of distal pancreatectomy have been implemented in the last two decades, besides perhaps the standardization of minimal invasive surgery; therefore we deemed that it has not a great impact on the outcomes. Second, despite adjusting for potential bias by using a rigorous propensity score-matching methodology, it is possible that unmeasured confounders were not accounted for, and sidelined as potential covariates. In spite of this, the AUC of our PS model was 0.847, indicating that the goodness-of-fit was very satisfactory which suggests that no important cofounders of the model were disregarded. In conclusion, we are aware that is the first step in the implementation of this novel technique, and a multicentric randomized clinical trial should be carried out to validate these results. In this regard, we are currently launching an RCT (clinicaltrials NCT04402346) to further strengthen our hypothesis.
